# Inhibition of hypertrophy and improving chondrocyte differentiation by MMP-13 inhibitor small molecule encapsulated in alginate-chondroitin sulfate-platelet lysate hydrogel

**DOI:** 10.1186/s13287-020-01930-1

**Published:** 2020-10-09

**Authors:** Shahrbanoo Jahangir, David Eglin, Naomi Pötter, Mojtaba Khozaei Ravari, Martin J. Stoddart, Ali Samadikuchaksaraei, Mauro Alini, Mohammadreza Baghaban Eslaminejad, Majid Safa

**Affiliations:** 1 Department of Tissue engineering & Regenerative Medicine, Faculty of Advanced Technologies in Medicine, Iran University of Medical Sciences, Tehran, Iran; 2 AO Research Institute Davos, Clavadelerstrasse 8, 7270 Davos, Switzerland; 3 Department of orthopedics and Trauma Surgery, Faculty of Medicine, Medical Center Albert-Ludwigs University, Albert-Ludwigs University of Freiburg, Freiburg im Breisgau, Germany; 4 Department of Stem Cells and Developmental Biology, Cell Science Research Center Royan Institute for Stem Cell Biology and Technology, ACECR, Tehran, Iran; 5 Cellular and Molecular Research Center, Iran University of Medical Sciences, Tehran, Iran; 6 Department of Medical Biotechnology, Faculty of Allied Medicine, Iran University of Medical Sciences, Tehran, Iran; 7 Department of Hematology, Faculty of Allied Medicine, Iran University of Medical Sciences, Tehran, Iran

**Keywords:** Chondrocyte hypertrophy, Platelet lysate, MMP-13 inhibitor, Small molecules, And mechanical properties

## Abstract

**Background:**

Mesenchymal stem cells are a promising cell source for chondrogenic differentiation and have been widely used in several preclinical and clinical studies. However, they are prone to an unwanted differentiation process towards hypertrophy that limits their therapeutic efficacy. Matrix metallopeptidase 13 (MMP-13) is a well-known factor regulated during this undesirable event. MMP-13 is a collagen degrading enzyme, which is also highly expressed in the hypertrophic zone of the growth plate and in OA cartilage. Accordingly, we investigated the effect of MMP-13 inhibition on MSC hypertrophy.

**Methods:**

In this study, 5-bromoindole-2-carboxylic acid (BICA) was used as an inhibitory agent for MMP-13 expression. After identifying its optimal concentration, BICA was mixed into a hydrogel and the release rate was studied. To prepare the ideal hydrogel, chondroitin sulfate (CS) and platelet lysate (PL) were mixed with sodium alginate (Alg) at concentrations selected based on synergistic mechanical and rheometric properties. Then, four hydrogels were prepared by combining alginate (1.5%w/v) and/or CS (1%w/v) and/or PL (20%v/v). The chondrogenic potential and progression to hypertrophy of human bone marrow-derived mesenchymal stem cell (hBM-MSC)-loaded hydrogels were investigated under free swelling and mechanical loading conditions, in the presence and absence of BICA.

**Results:**

Viability of hBM-MSCs seeded in the four hydrogels was similar. qRT-PCR revealed that BICA could successfully inhibit MMP-13 expression, which led to an inhibition of Coll X and induction of Coll-II, in both free swelling and loading conditions. The GAG deposition was higher in the group combining BICA and mechanical stimulation.

**Conclusions:**

It is concluded that BICA inhibition of MMP-13 reduces MSC hypertrophy during chondrogenesis.

**Graphical abstract:**

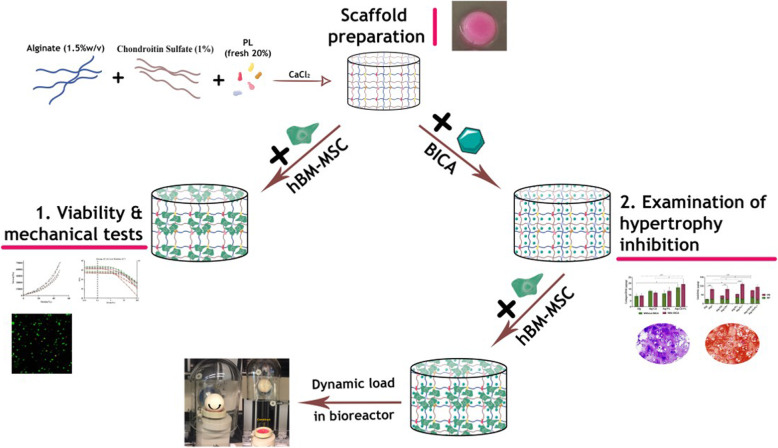

## Introduction

Articular cartilage is a highly organized tissue with poor self-healing ability caused by its avascular and aneural structure [1–3]. Accordingly, cartilage defects are extremely common due to age-related problems, trauma, or osteoarthritis (OA), leading to compromised cartilage functionality [4, 5].

Available therapies have not yet been able to fully restore the biological and biomechanical function of damaged cartilage [6]. Despite the rapid developments regenerative medicine and tissue engineering have experienced in recent years, promising clinical applications for cartilage regeneration are still lacking. The use of an appropriate cell source is a key factor in tissue engineering. Bone marrow-derived mesenchymal stem cells (BM-MSCs) have been widely used in cartilage tissue engineering due to their relative ease of isolation, proliferation capacity, and chondrogenic potential [7–14]. However, it has been shown that BM-MSCs tend to acquire a hypertrophic phenotype, which is characterized by the upregulation of Coll X and MMP-13 [15, 16]. The progression of MSCs towards a hypertrophic phenotype is a major obstacle for the optimal clinical use of MSCs for cartilage repair.

Among the biomarkers of chondrocyte hypertrophy, Coll X and MMP-13 are the most widely used [17]. Coll X is normally expressed in the hypertrophic region of growth plate, but not in healthy cartilage; however, it is overexpressed at the protein and mRNA levels in OA human cartilage [18, 19]. MMP-13 is an ECM-degrading enzyme, which is also highly expressed in the hypertrophic zone of the growth plate and in OA cartilage [17, 20].

In the present study, we investigated the effect of MMP-13 inhibition on MSC hypertrophy. There are different ways to directly or indirectly suppress MMP-13 expression, such as small molecules or microRNAs (miRNAs) [21–23]. Small molecules, as stable and cost-effective agents [24], have been employed in different clinical therapies [25].

Several types of MMP-13 inhibitor for OA and cancer treatment have been investigated. Although they showed good initial results, but they were unsuccessful in clinical trials due to the off-target effects and musculoskeletal syndrome after injection [26, 27]. Therefore, using a construct for localized delivery of MMP-13 inhibitors into the lesion may provide prolonged drug exposure and limit their side-effects. Accordingly, within this study, we investigated the effect of the small molecule BICA loaded in a hydrogel on MMP-13 inhibition during BM-MSC chondrogenesis.

Alginate is a hetero-polysaccharide made of linked β-d-mannuronic acid and α-l-guluronic acid monomers, extracted from brown sea algae [28–30]. Alg has been extensively studied in biomedical applications especially in tissue engineering owing to the biocompatibility, non-toxicity, non-immunogenicity, good gel forming ability, and low cost [28, 29, 31]. Previous studies indicated Alg hydrogel provides an optimal milieu for chondrogenic differentiation of encapsulated MSCs [30, 32–34]. However, Alg has shortcomings that include absence of cell adhesion motifs and weak mechanical properties [31, 35–37]. Therefore, it is often used in combination with other materials such as chitosan [38], hyaluronic acid [39, 40], chondroitin sulfate [41], and synthetic polymers (poly (lactic acid)) [31, 42] to improve its mechanical and biological properties.

Chondroitin sulfate is a glycosaminoglycan made by repeating disaccharide unit of d-glucuronic acid and *N*-acetyl galactosamine sulfate. CS, as a main component of cartilage extracellular matrix, plays an important role in modulation of inflammation, control of cartilage metabolism, intracellular signaling, linking extra cellular matrix (ECM) with cell surface proteins, and cartilage phenotype regulation [35, 43, 44]. It has been shown that CS improves MSC chondrogenesis in hydrogels [45]. In addition, the interaction of CS with Alg increases negative charge, providing more space for cell growth and differentiation within the composite hydrogels [46].

Platelet lysate is a cocktail of growth factors, anti-inflammatory cytokines, and clot-forming proteins that are involved in tissue repair processes [47, 48]. PL is a cost-effective and autologous source of growth factors compared to recombinant growth factors [49]. Studies suggest that PL significantly increases MSC proliferation compared with fetal bovine serum (FBS) in vitro. It can also enhance the proliferative capacity of senescent MSCs [50, 51]. In addition, PL enhances chondrogenic differentiation of MSCs [52]. However, there are two main drawbacks for PL as scaffold in tissue engineering: a high degradation rate and low mechanical properties [49, 53]. Therefore, the mechanical stability of PL-based hydrogels for long-term applications needs to be improved. In this study, we aimed to incorporate PL and CS within an Alg gel to create a hydrogel matrix combining the biological and mechanical properties of the different materials.

Furthermore, it has been shown that mechanical loading has a paramount effect on the development of articular cartilage [54]. Chondrogenesis of MSCs has been demonstrated under multiaxial loading in fibrin-polyurethane scaffolds [55–57]. In addition, it has been observed that the multi-complex mechanical forces, like shear, compression, and torque were able to regulate the expression of MMPs [58–60]. Therefore, we exposed our constructs to a multiaxial load bioreactor, to examine the effect of mechanical load on chondrogenic differentiation of hBM-MSCs.

## Materials and methods

### Materials

5-Bromoindole-2-carboxylic acid (B2761), MTT powder (M2128), α-MEM Powder, alginate sodium (Alg, A2033), chondroitin sulfate (CS, C6737), calcium chloride (CaCl2, 55670), phosphate buffer saline (PBS, P4417), calcein AM (17783), ethidium homodimer-1 (46043), and agarose (A0169) were obtained from Sigma, FBS (Pan Biotech, Aidenbach, Germany).

### Selection of the best CS and PL concentration

For choosing the best concentration of CS, we prepared the hydrogel by blending a 1.5% (w/v) Alg with three different concentrations of CS 0.5, 1, and 2.5% (w/v), and then, the hydrogels were incubated in agarose mold wells (6 mm Ø × 3 mm H) at RT. The agarose molds were prepared from 3% w/v agarose in CaCl_2_ 150 mM for in situ gelling of the Alg solution. The obtained gels were kept overnight in a solution of 150 mM CaCl_2_ before using for evaluation of mechanical properties by compression testing. The hydrogels were also prepared in a larger agarose mold of 25 mm Ø × 2 mm H, for rheology tests.

We also prepared the Alg hydrogel with different concentrations of fresh and freeze-dried PL. Briefly, three different concentrations of fresh PL (10, 20, and 50% (v/v)) and also three different concentrations of freeze-dried PL (0.5, 1, and 2% (w/v)) were mixed with 1.5% (w/v) sodium alginate, then cast into the agarose mold, covered with 150 mM CaCl_2_, and incubated at RT.

Preparation of the PL was performed as follows:

Blood samples (300 ml) were collected after obtaining written consent from healthy volunteers according to the ethical approval from Ethics Committee of Royan institute (IR.ACECR.ROYAN.REC.1395.174). The samples were centrifuged at 200*g* for 30 min at RT. Then, the resulting plasma supernatants were pooled, transferred into a 15-mL Falcon tube, and centrifuged at 2000*g* for 5 min at RT to produce a platelet pellet. The platelet pellet was resuspended in PBS (1/10th of the initial blood volume), sonicated for 15 min at RT, and then stored at − 20 °C to use later as PL.

#### Compression mechanical test

Unconfined hydrogels were compressed using an Instron 5866 electromechanical test device equipped with a static load cell of 100 N, at a displacement of 1 mm/min and stopped at 50% sample’s height (*n* = 6). Young’s modulus was calculated according to the stress-strain-curve.

#### Rheological analysis

The viscoelastic properties of the various hydrogel formulations were investigated using an Anton Paar MCR-302 rheometer equipped with a Peltier temperature control unit and cone-plate geometry (Disposable Measuring Plate PP25 with a 25-mm diameter). A fixed gap of 0.8 mm was selected, and a time sweep was conducted at 100% strain at 25 °C. The storage modulus (G′) and loss modulus (G″) values were calculated at 0.1% strain (*n* = 6).

### Hydrogel preparation and characterization

Hydrogel was prepared by mixing alginate sodium solution, CS, and PL to a final concentration of 1.5% (w/v) Alg, 1% (w/v) CS, and 20% (v/v) PL. The aqueous hydrogel mixture was cast into the agarose mold, covered by 150 mM calcium chloride (CaCl_2_), and incubated at room temperature (RT) overnight before characterization. The detailed hydrogel formulations are shown in Table 1.

**Table 1:** List of hydrogel formulations

Hydrogel description	Alginate (%w/v)	Chondroitin Sulfate (%w/v)	PL
Alg	1.5	0	0
Alg-CS	1.5	0.5, 1, 2.5	0
Alg-PL	1.5	0	Freeze-dried: 0.5, 1, 2 (%w/v)
			Fresh: 10, 20, 50 (%V/V)
Alg-CS-PL	1.5	1	Fresh: 20(%V/V)

The mechanical properties of cell-free and BM-MSC embedded hydrogels were assessed. hBM-MSCs were embedded in hydrogels (5 × 10^6^/ml), and the mixture was incubated in a solution of 150 mM CaCl_2_ for 1 h prior to mechanical analysis (*n* = 3).

### Determination of ideal concentration of BICA

#### Cell viability evaluation

Cell toxicity of BICA was evaluated in duplicate using MTT assay at two time points (days 3 and 7). hBM-MSCs were isolated from bone marrow samples (2 donors aged 56 and 60 years old) according to the ethical approval from Ethics Committee of Royan institute (IR.ACECR.ROYAN.REC.1395.174). Briefly bone marrow aspirates were harvested during routine procedures from each patient’s iliac crest; after isolation, hMSCs were subcultured an initial cell density of 1 × 10^4^ cells/cm^2^ in α-MEM supplemented with 15% fetal bovine serum (FBS) and 100 mg/mL penicillin–streptomycin. The cells were expanded through subcultures until passage 3 to use for other procedures. The cells were also characterized in terms of trilineage differentiation and expression of some surface markers (CD44, CD73, CD105, CD90, CD11b, CD45, and CD34). hMSCs were cultured in a 24-well plate at a density of 1 × 10^4^ cell/cm^2^ in α-MEM supplemented with 10% FBS and six different concentrations of BICA (1, 10, 20, 31, 39, and 50 μM). Cells were washed with PBS followed by removing the culture medium, then incubated in 0.5% of 3-(4,5-dimethylthiazol-2-yl)-2,5-diphenyltetrazolium bromide (MTT) for 2 h at 37 °C in a 5% CO2 atmosphere incubator. MTT was replaced with an equal volume of DMSO to dissolve the formazan crystals. Absorbance was measured at 540 nm by a Multiskan Spectrum microplate reader (ThermoScientific, USA).

#### Effect of BICA on chondrogenesis of hBM-MSC

In order to investigate the effect of BICA on chondrogenic differentiation, standard pellet culture method was used [61]. Briefly, hBM-MSC pellets were formed by centrifuging 2.5 × 10^5^ hBM-MSCs at 300*g* for 5 min in chondrogenic culture medium; high glucose DMEM supplemented with ITS (1%), ascorbic acid (50 μg/ml), non-essential amino acid (1%), Dexamethasone (10^− 7^ M), l-glutamine (2 mM), and TGF-β1 (10 ng/ml). Six different concentrations of BICA, as above, were added to the chondrogenic medium. A control group without BICA was also prepared. The medium was changed every 3 days until the pellets were harvested at day 28 for further analysis. The collected media were also pooled and kept at 4 °C until further investigation.

##### Biochemical analysis for glycosaminoglycan, collagen, and DNA content assay

Glycosaminoglycan (GAG) assay was performed on intact pellets (INT) and the conditioned media (CM). Pellets were rinsed with PBS and digested overnight in proteinase-K (0.5 mg/ml) at 56 °C. DMMB (1,9-dimethyl-methylene blue) was used in order to quantify the sulfated glycosaminoglycan. Briefly, 200 μl of DMMB color reagent was added to 20 μl of sample or chondroitin sulfate standard. The absorbance was measured immediately at 535 nm. The results were normalized to the DNA content quantified by PicoGreen assay on the same proteinase-K digest as above. For this purpose, PicoGreen was added to each sample or DNA standard and incubated at RT for 5 min. The fluorescence was measured at excitation 485 nm and emission 535 nm. On the same digested samples, the collagen content was evaluated with Sircol™ Insoluble Collagen Assay kit, according to the manufacturer’s instructions. Briefly, 1 ml Sircol dye reagent was added to 50 μl of sample or standard and gently mixed on a mechanical shaker for 30 min. The tubes were drained followed by centrifugation at 12,000 rpm for 10 min. The pellet was dissolved in Alkali reagent after removing the unbound dye with an ice-cold Acid-Salt Wash reagent. The absorbance was recorded at 550 nm, and the results were normalized to the DNA content. All absorbance/fluorescence measurements were performed by a Victor3 micro plate reader. GAG, Collagen, and DNA assays were done in triplicate.

##### Real-time PCR analysis

The pellets were washed with PBS and snap frozen in liquid nitrogen. Total RNA was extracted using TRI Reagent (Molecular Research Center TR-118, Cincinnati, USA). Reverse transcription was performed with random hexamer primers and TaqMan reverse transcription reagents. All real-time quantitative polymerase chain reactions (PCR) for the pellet culture studies were performed with a SYBR Premix Ex TaqTM II (TaKaRa RR820L, Kusatsu, Japan) on an ABI StepOnePlusTM Q-PCR system (applied Biosystems Life Technologies) for Coll X and MMP-13 genes. The expression level of target genes was normalized to β-actin as the reference gene. The analysis was performed by the comparative ∆∆CT method. Primers are listed in Table S1(supplementary material).

##### Histological evaluation

hBM-MSC pellets were fixed in 4% paraformaldehyde and incubated in 4 °C for 3 days. After processing in a tissue preparation machine (Did Sabz co, Iran), the samples were embedded in paraffin. Six-micrometer sections were stained with safranin O and fast green and toluidine blue.

### Release kinetics of BICA in tri-part hydrogels

In order to examine the kinetic release of BICA, alginate-based hydrogels were used. The best concentration of BICA was added into four hydrogels with different compositions (Table 1). The hydrogels were prepared in the cap of a 1.5-mL Eppendorf tube and cross-linked with a 150 mM CaCl_2_ solution at RT for 1 h. The remaining solution was stored to calculate the amount of non-encapsulated drug. The BICA-embedded hydrogels were incubated in 1 ml PBS at 37 °C (*n* = 3). Aliquots from the 1 ml PBS solution were collected and replaced with 1 ml fresh PBS at specific time points, and the BICA concentration was determined by a Thermo Scientific™ Multiskan™ GO Microplate Spectrophotometer (Thermo Scientific™, USA) at a wavelength of 290 nm.

### Embedded hydrogels with mesenchymal stem cells

Human BM-MSCs were isolated from bone marrow samples (3 donors aged 15, 18, and 37 years) after ethical approval from the cantonal ethical commission of Bern (KEK: Req-2016-00141) using previously described protocols [62]. Bone marrow samples were aspirated of vertebral bodies from each donor, after isolation, at 70–80% confluence, hBM-MSCs were harvested by trypsinization and subcultured at a density of 3 × 10^3^ cells/cm^2^ in Minimum Essential Medium supplemented with 10% Sera Plus bovine serum, 100-U/mL penicillin, and 100-mg/mL streptomycin, and 5 ng/ml FGF and grown until passage 3. Cultures were maintained at 37 °C/5% CO^2^, and the medium was refreshed every second day. hBM-MSCs were also seeded into the hydrogels at a density of 5 × 10^6^ cell/ml at passage 3. The cell-laden hydrogels were cast into a mold (8 mm Ø × 5 mm H) and incubated in 150 mM CaCl_2_ for 1 h at RT. Then, washed with PBS and incubated in chondrogenic culture medium for more analysis.

#### Live and dead assay

After embedding hBM-MSCs into the Alg-based hydrogels, the cell-laden constructs were cultured in αMEM supplemented with 10% v/v SeraPlus, 1% v/v of Pen/Strep, and 5 ng/ml recombinant human fibroblast growth factor-2 (FGF-2). Then, cell viability was assessed at 2 time points (days 3 and 7) on the bulk hydrogel, using live/dead assay, where living cells were stained with Ca-AM and dead cells with EthD-1. At each time point, the hydrogels were removed from the culture medium and incubated in a staining solution containing 5 μM Ca-AM and 1 μM EthD-1 prepared in serum-free DMEM LG for 1 h at 37 °C, within a humidified atmosphere of 5% CO_2_. After incubation, cells were imaged with confocal laser scanning microscopy (LSM810, Zeiss). For each sample, single plane and Z stack (1300 μm) were acquired and tile scans were generated to image a larger sample area. Four different fields of view per sample were used to quantify cell viability by counting the red (dead) and green (viable) cells in Image J software.

### Chondrogenesis assessment

Chondrogenic differentiation of cell-laden hydrogels (with and without 39 μM BICA) was investigated by qRT-PCR, biochemical assessment, and histological staining after 28 days. Total RNA was isolated using TRI Reagent® Solution (Molecular Research Centre Inc., Cincinnati, OH, USA) according to the manufacturer’s protocol. Reverse transcription of 1 μg total RNA was performed by TaqMan Reverse Transcription Kit (Applied Biosystems, Foster City, USA). Relative gene expression (quantitative polymerase chain reaction (qPCR)) reactions were set up in 10-μL reaction mixtures containing TaqMan Universal Master Mix (Thermo Fisher, Zürich, Switzerland), the appropriate set of primers and probes, DEPC-H_2_O and cDNA template. For gene expression, Coll I, Coll II, ACAN, ALP, Coll X, and MMP-13 were evaluated, and 18S was used as a housekeeping gene. The expression of genes was normalized to a control of encapsulated hBM-MSCs into Alg- hydrogel, incubated for 24 h. Technical triplicates were used for each target gene and for the different donors (*n* = 3). Primer and probe sequences are shown in supplemental Table S2 (supplementary material), while catalog numbers of Assays-on-Demand (Applied Biosystems, Foster City, USA) are listed in the supplemental Table S3 (supplementary material). GAG and collagen content in the samples were normalized to DNA content. In addition, GAG in the conditioned medium was also measured. Histological staining analysis was performed.

### The effect of mechanical loading on the chondrogenesis of hBM-MSCs in hydrogels

Chondrogenesis of hBM-MSCs in the best hydrogel (Alg-CS-PL with and without 39 μM BICA) was evaluated in the presence or absence of mechanical loading. The mechanical loading was produced by multiaxial loading bioreactor [63]. Briefly, a ceramic ball (32 mm in diameter) was pressed onto the scaffold. An interfacial shear load was generated by ± 25° oscillatory rotation of the ball about the axis perpendicular to the scaffold’s axis (at a fixed indentation of 0.2 mm with a frequency of 0.1 Hz). After an initial 2-week culture without loading, mechanical stimulation was applied 1 h per day for five consecutive days per week over a period of 2 further weeks. After a total of 4 weeks’ culture, samples were harvested for qRT-PCR, histology, and biochemical analyses (2 different donors with 2 replicates).

### Statistical analysis

Data were obtained from the samples and represented as the mean ± standard deviation (SD). Statistical assessment was carried out by analysis of two-way ANOVA and post hoc Tukey’s tests. Statistical analysis was performed by means of Prism software (GraphPad Software, La Jolla, CA, USA).

## Results

### Determination of CS and PL concentration for combination with Alg hydrogel

**Fig. 1:**
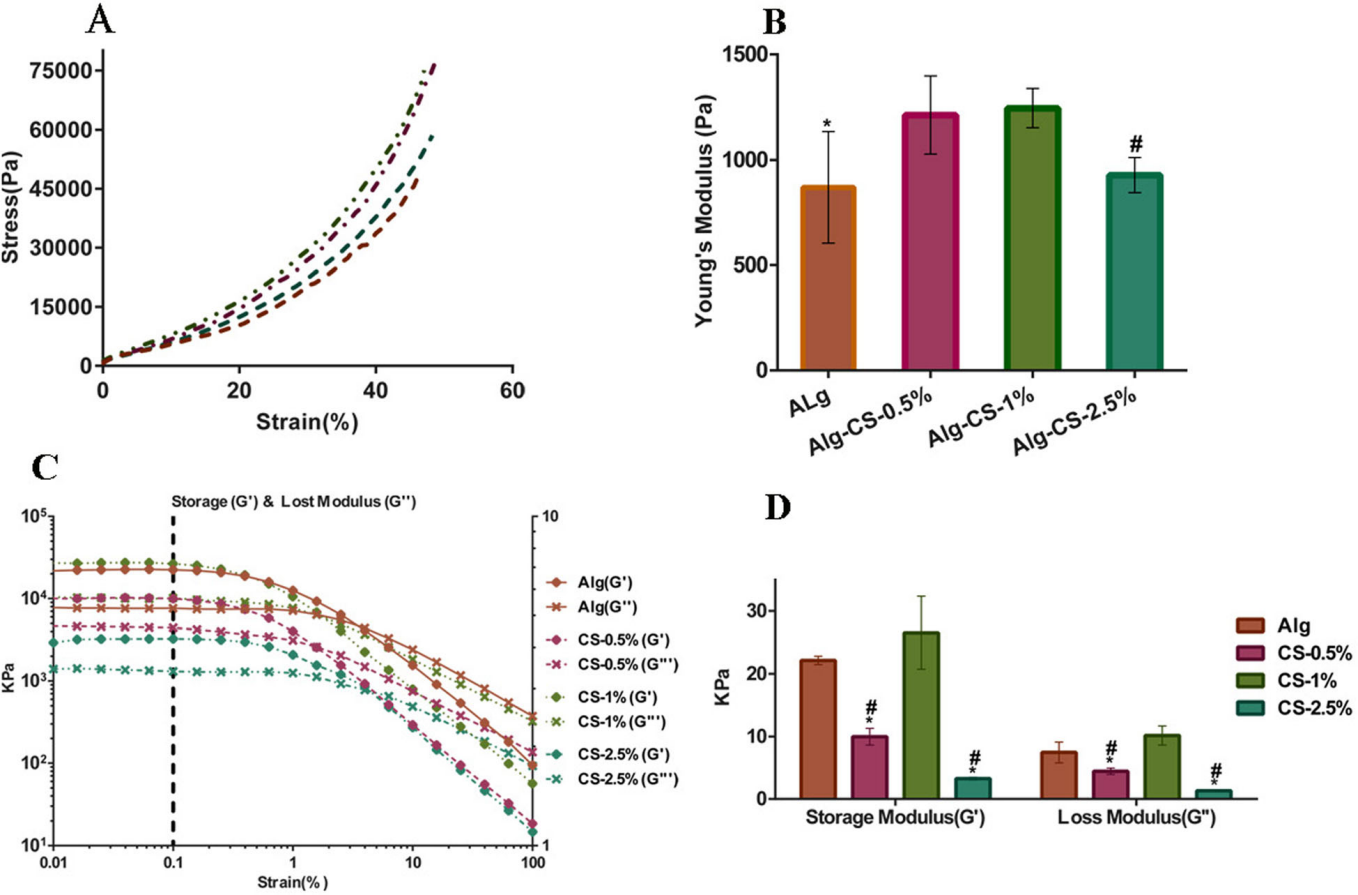
Mechanical properties of hydrogels with different concentrations of CS. **a** Compressive stress-strain curve. **b** Young’s modulus. The results showed higher Young’s modulus in the Alg-CS-1% compared with Alg and Alg-CS-2.5% hydrogels (*, ^#^ presented a statistically significant differences compared to Alg-CS-1% with *P* ≤ 0.01, *P* ≤ 0.05 respectively). **c** Rheological measurements. **d** G′ and G″ calculated at 0.1% strain. The elastic (G′) and viscous (G″) modulus were significantly higher in the Alg-CS-1% hydrogel compared with Alg hydrogels containing CS (0.5, 2.5%). The Alg hydrogels with CS 0.5% and 2.5% had significantly lower G′ and G″ compared with Alg hydrogel (*, ^#^ presented a statistically significant difference compared to Alg-CS-1% and Alg hydrogels, respectively (*p* ≤ 0.001)) (*n* = 6)

Mechanical testing and rheological measurements were used to select the best concentration of CS and PL to incorporate with Alg. Figure 1a shows compressive stress-strain curves for Alg hydrogel (1.5% w/v) with 3 different concentrations of CS (0.5, 1, 2.5% w/v). The Young’s modulus calculated from stress-strain curves (Fig. 1b) shows a significant increase in the Alg hydrogel with CS-1% (1245.7 Pa ± 92.45) compared with Alg hydrogel alone (869.1125 Pa ± 265.52) (*P* ≤ 0.01) and Alg incorporated with CS-2.5% (927.724 Pa ± 83.46) (*P* ≤ 0.05).

Figure 1c indicates the rheological measurement of hydrogels with three concentrations of CS, while Fig. 1d shows the G′ and G″ calculated at 0.1% strain of rheometer assessment. Our results demonstrated that the Alg hydrogel with CS-1% had significantly higher G′ and G″ compared with 0.5 and 2.5% CS hydrogels (*p* ≤ 0.001) (Fig. 1d). Furthermore, the hydrogels with 0.5 and 2.5% CS showed significantly lower G′ and G″ in comparison to the Alg hydrogel alone (*P* ≤ 0.001). To this end, Alg hydrogel with CS-1% was selected for further investigation.

**Fig. 2:**
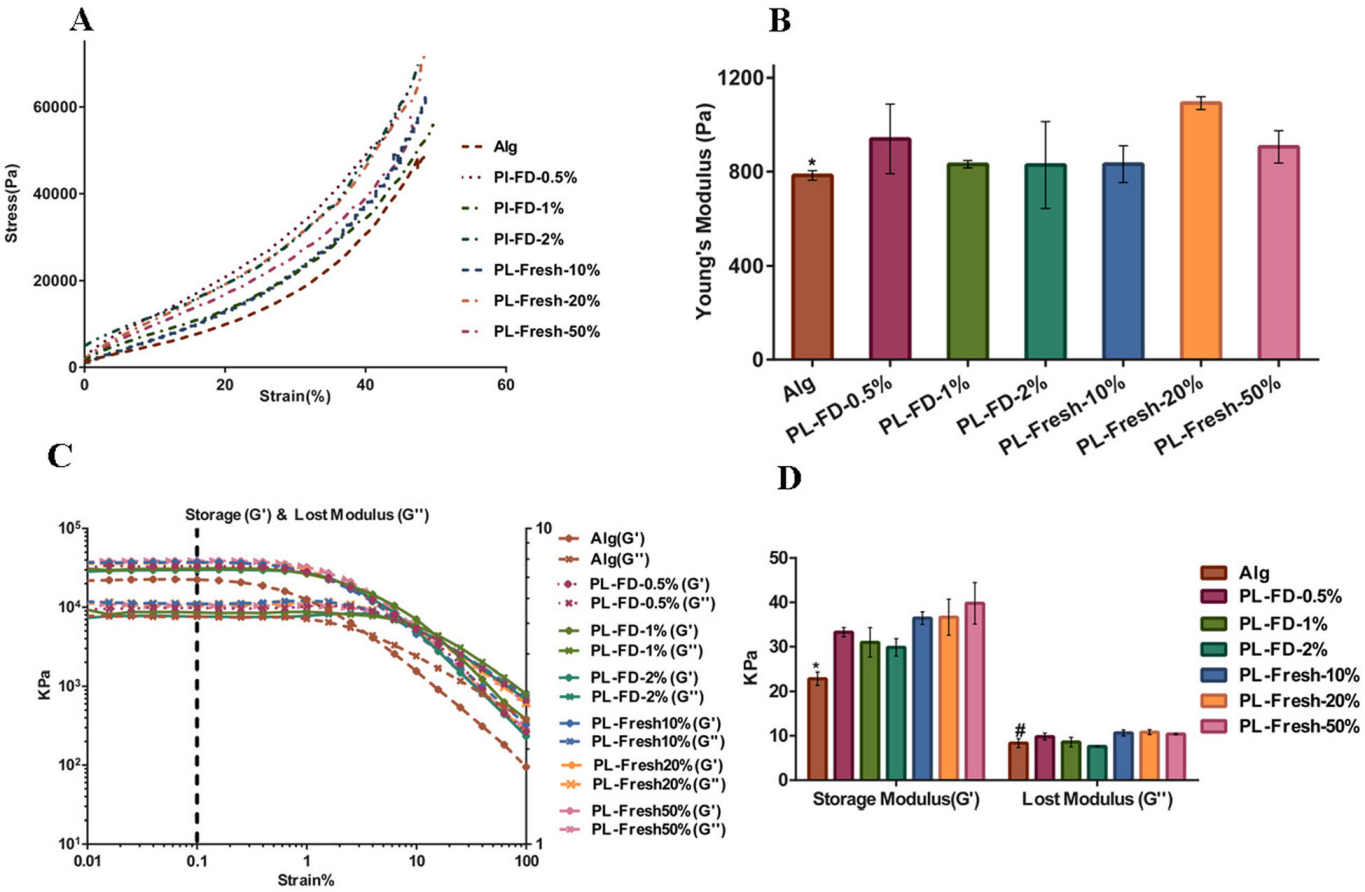
Mechanical properties of hydrogels with different concentrations of PL. **a** Compressive stress-strain curve. **b** Young’s modulus calculated from A curves shows a significant increase in Alg with fresh-PL-20% (v/v) hydrogel compared with Alg hydrogel alone (* *P* ≤ 0.05). **c** Rheological measurements. **d** G′ and G″ calculated at 0.1% strain. The results showed significant higher elastic (G′) modulus in the Alg-PL hydrogels (all concentrations) compared with Alg hydrogel alone (* *p* ≤ 0.01), and viscous (G″) modulus were significantly higher in all the Alg-PL hydrogels (fresh ones) compared with Alg hydrogel alone (^#^*p* ≤ 0.05) (*n* = 6)

Figure 2a shows compressive stress-strain curves for Alg hydrogels with three different concentrations of freeze-dried PL and three different concentrations of fresh PL. Figure 2b shows a significantly higher Young’s modulus in the Alg with PL-fresh-20%(v/v) (1092.436 Pa ± 27.408) compared with Alg hydrogel (783.99 Pa ± 20.18) (*P* ≤ 0.05). There was no statistically significant difference between other groups. In addition, Fig. 2c shows the rheological measurement of hydrogels with different concentrations of PL (freeze-dried and fresh), and Fig. 2d shows the G′ and G″ calculated at 0.1% strain of rheometer assessment. The results indicated that hydrogels with different concentrations of PL, both fresh and freeze-dried, had significantly higher G′ compared with Alg hydrogel alone (*P* ≤ 0.01).

Similarly, hydrogels at all concentration of fresh PL showed significantly higher G″ in comparison to the Alg hydrogel (*P* ≤ 0.05). There was no significant difference in G″ between freeze-dried PL and Alg hydrogel. The fresh PL 20% was selected for further investigation based on the Young’s modulus value (1092.436 Pa ± 27.408).

### Mechanical compression analysis of alginate-based hydrogels containing CS and PL

**Fig. 3:**
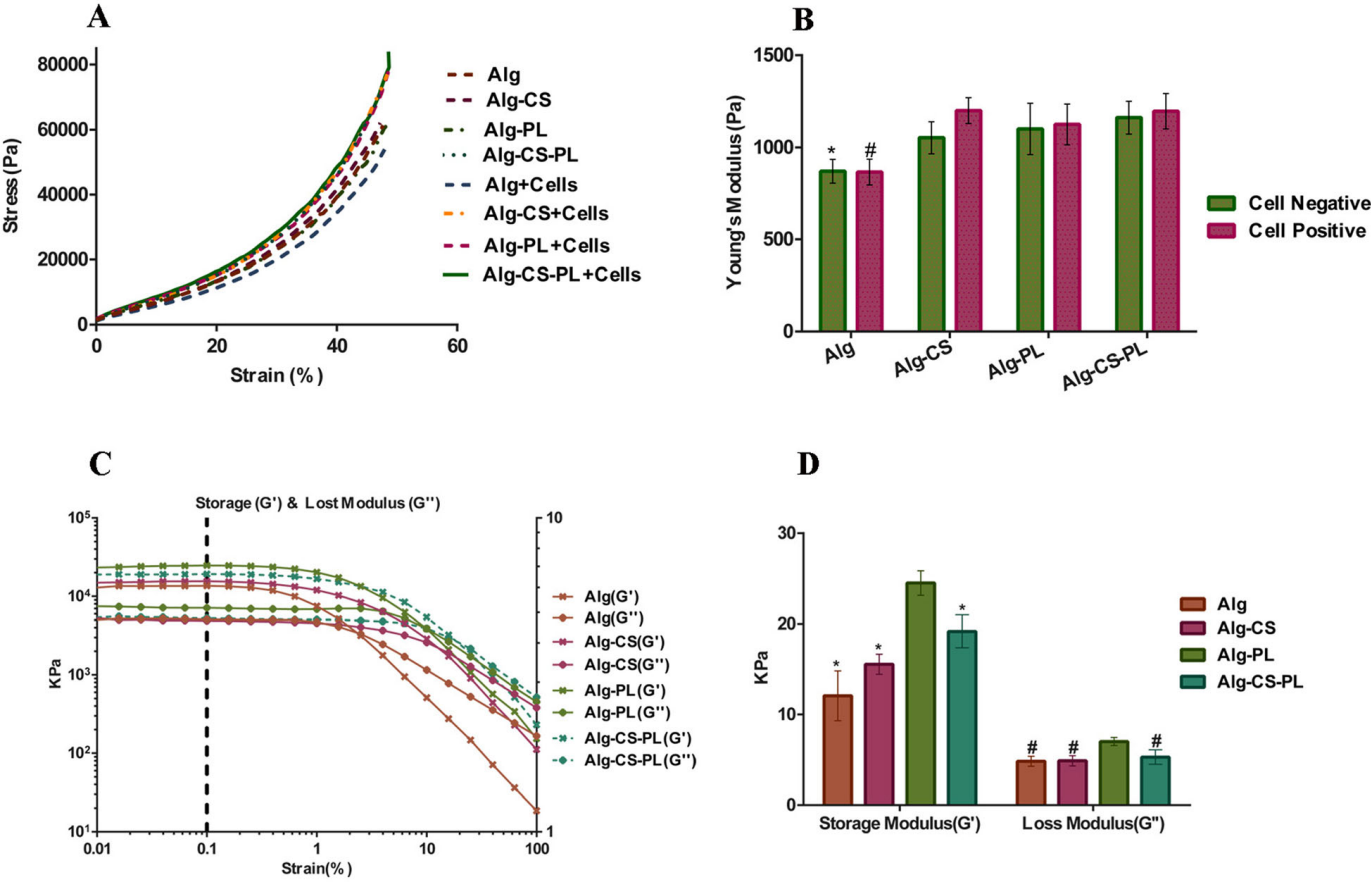
Mechanical properties of different hydrogels with or without cells. **a** Compressive stress-strain curve. **b** Young’s modulus calculated from the stress-strain curve. The chart reveals a significantly higher Young’s modulus in the Alg gel containing either both fresh-PL-20% /CS1% or only one of them when compared to Alg hydrogel with or without cells. No significant difference was observed in cell free hydrogels compared with cell containing hydrogels (*, ^#^ presented a statistically significant difference compared to other groups in both conditions with or without cells (*p* ≤ 0.05)). **c** Rheological measurements of four different hydrogels. **d** Calculated elastic (G′) and viscous (G″) modulus at 0.1% strain. The results indicated significantly higher G′ and G″ in the Alg-PL hydrogel compared with the other hydrogels (* (*p* ≤ 0.01), ^#^ (*p* ≤ 0.05)) (*n* = 3)

Three different hydrogels were prepared by incorporating of CS (1% w/v), PL (20% v/v), or both with Alg hydrogel (1.5% w/v). An unmodified Alg hydrogel was used as a control. Figure 3a shows hydrogel strain-stress curves, while Young’s modulus is shown in Fig. 3b. The results indicated a significant increase of Young’s modulus in Alg incorporated CS, and PL or both compared with Alg alone (*p* ≤ 0.05). No significant difference between cell laden hydrogels and hydrogels without cells was observed.

The viscoelastic properties of the alginate-based hydrogels were also assessed. The G′, G″ versus strain are shown in Fig. 3c. The results showed at 0.1% strain higher G′ (*p* ≤ 0.01) and G″ (*p* ≤ 0.05) in Alg-PL hydrogels in comparison to the other hydrogels (Fig. 3d).

### Selecting the effective concentration of BICA

**Fig. 4:**
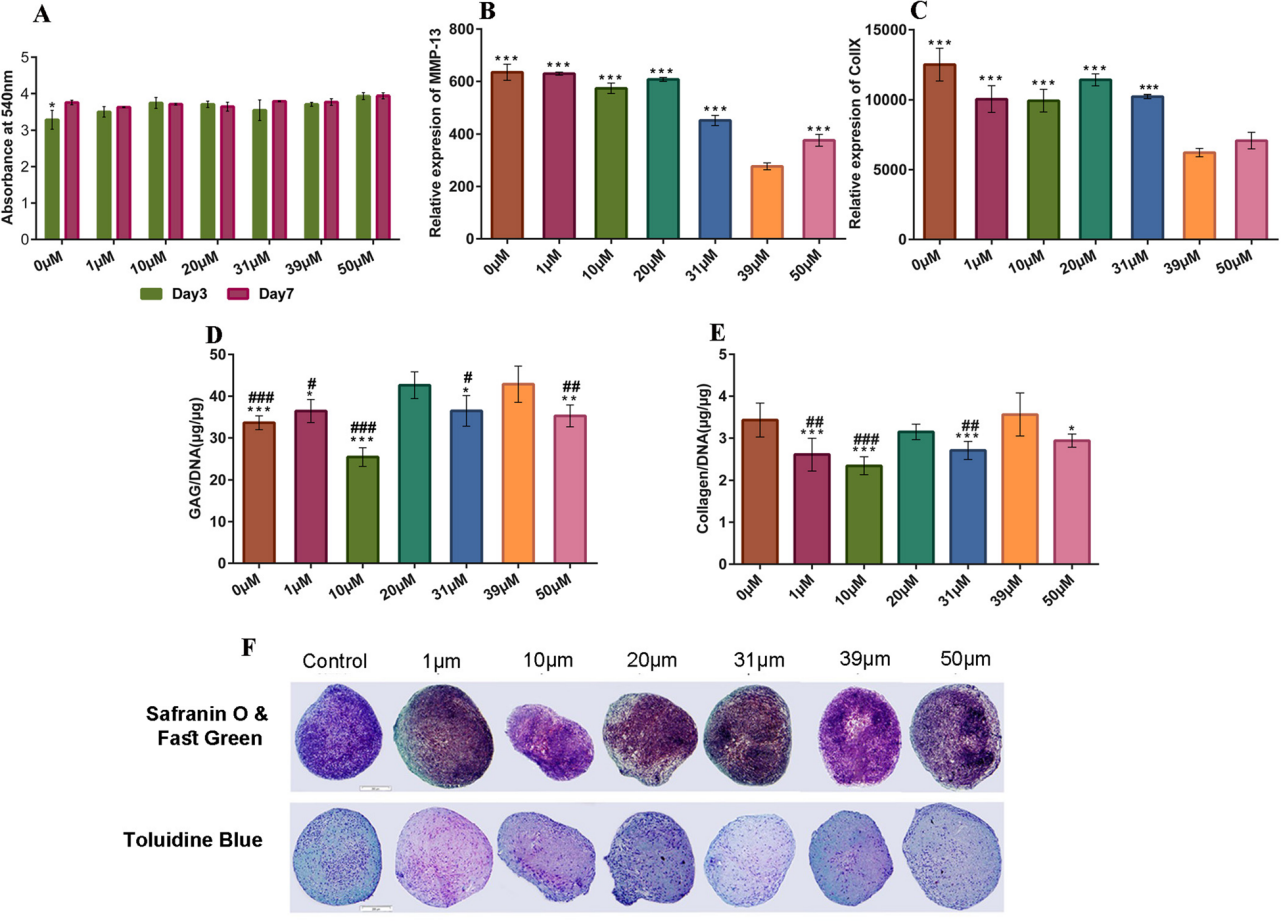
Selecting the effective concentration of BICA. **a** MTT assay showed no adverse effect at any concentration of BICA on the metabolic activity of the hBM-MSCs cultured in pellets for 3 and 7 days. **b** After 28 days of chondrogenic pellet culture, the expression of MMP-13 gene at 39 μM was significantly lower than the other groups (***P ≤ 0.001). **c** The expression of Coll X gene at 39 μM was significantly lower than all groups, except for 1 μM and 50 μM (***P ≤ 0.001). **d** GAG production was significantly higher at 20 and 39 μM compared to the other groups (*, ^#^ indicate a significant difference against 39 μM and 20 μM respectively). **e** Total collagen content was significantly higher at 39 μM (and control) compared to the other groups (*, ^#^ indicate a significant difference against 39 μM and control respectively). **f** Toluidine blue and Safranin-O and fast green staining of different concentrations of BICA on MSC pellet culture

The cytotoxicity of BICA in monolayer was investigated by MTT assay. The results indicated that all the tested concentrations of BICA had no adverse effect on the metabolic activity of hBM-MSCs at defined times (days 3 and 7). Figure 4a shows the absorbance of formazan-dissolved solution at 540 nm in control and treated groups.

The effect of BICA on chondrogenesis was investigated in pellet culture over 28 days. The gene expression level of two hypertrophy-associated genes (MMP-13 and Coll X) was evaluated by qRT-PCR. The relative expression of MMP-13 showed a significant decrease at 39 μM BICA compared to the other groups (*P* ≤ 0.001) (Fig. 4b). Similarly, the lowest expression of Coll X was also observed at the BICA concentration of 39 μM (*P* ≤ 0.001) (Fig. 4c).

GAG and collagen content of hBM-MSC pellets that were cultured with BICA was also evaluated. At BICA concentrations of 20 μM and 39 μM, GAG secretion was greater than the control group (*P* ≤ 0.001) (Fig. 4d). The highest collagen content was observed at 39 μM BICA, which was significantly compared to other groups except for the control group and 20 μM BICA (Fig. 4e). Histological staining was performed on pellets with different concentrations of BICA. Chondrogenic differentiation was observed in hBM-MSC pellets cultured in chondrogenic media with and without BICA (Fig. 4f). Following these results, the concentration of BICA of 39 μM was selected for further studies.

### In vitro release study

**Fig. 5:**
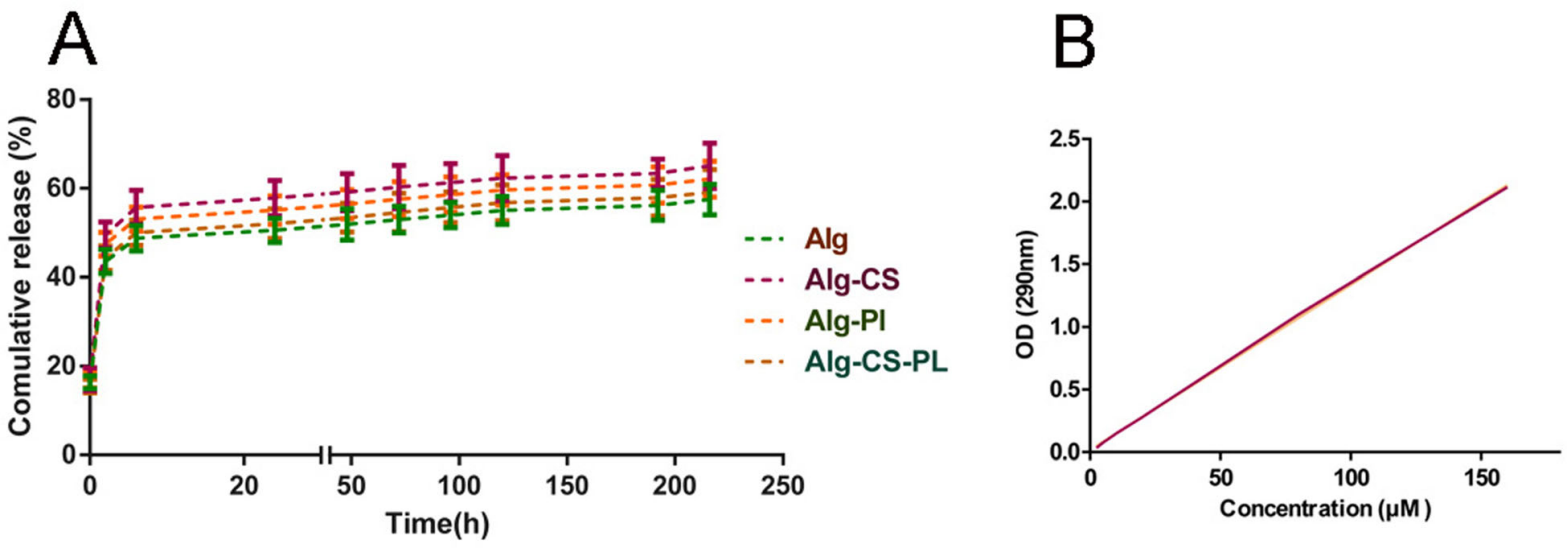
**a** Cumulative release rate of BICA in hydrogels. The sustained release pattern is similar for all hydrogels (*n* = 3). **b** Standard curve for BICA at 290 nm

In vitro release of BICA from hydrogels was studied for 9 days. The release profile showed a burst release of 28–33% of BICA within the first 2 h for all groups. Then, a similar sustained release profile was observed for all groups. After 72 h, between 36 and 43% of BICA were released from all the 4 hydrogels. A near-plateau profile was observed for all groups until day 9. (Fig. 5a and b).

### Live-dead assay for cell containing alginate-based hydrogels

**Fig. 6:**
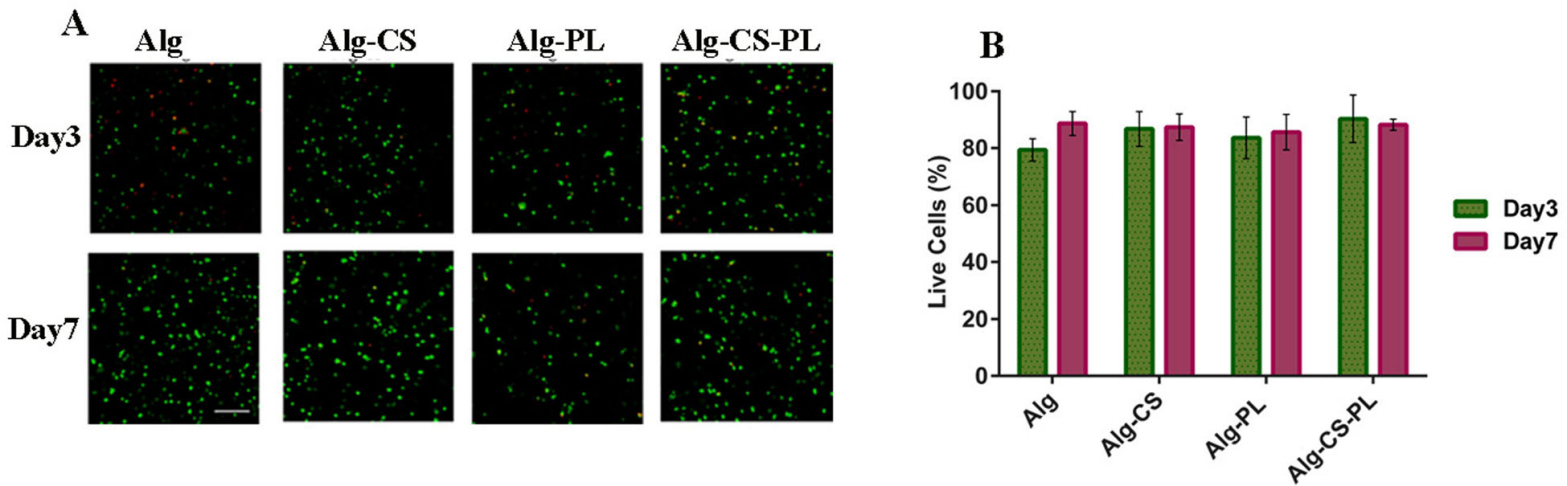
Live-dead assay staining. **a** Live (green) and dead (red) cells in four different hydrogels at days 3 and 7 (scale bar = 100 μm). **b** Cell viability percentage at days 3 and 7

To evaluate whether the crosslinked Alg-based hydrogels affect cell viability, we encapsulated hBM-MSCs within different hydrogels and cultured them for 1 week. Hydrogel constructs were then stained with Live-Dead dye staining at 2 time points (day 3 and 7). As shown in Fig. 6a and b, no decrease in cell viability was observed in the different hydrogels.

### Differentiation assessment for cell laden hydrogels

#### qRT-PCR

**Fig. 7:**
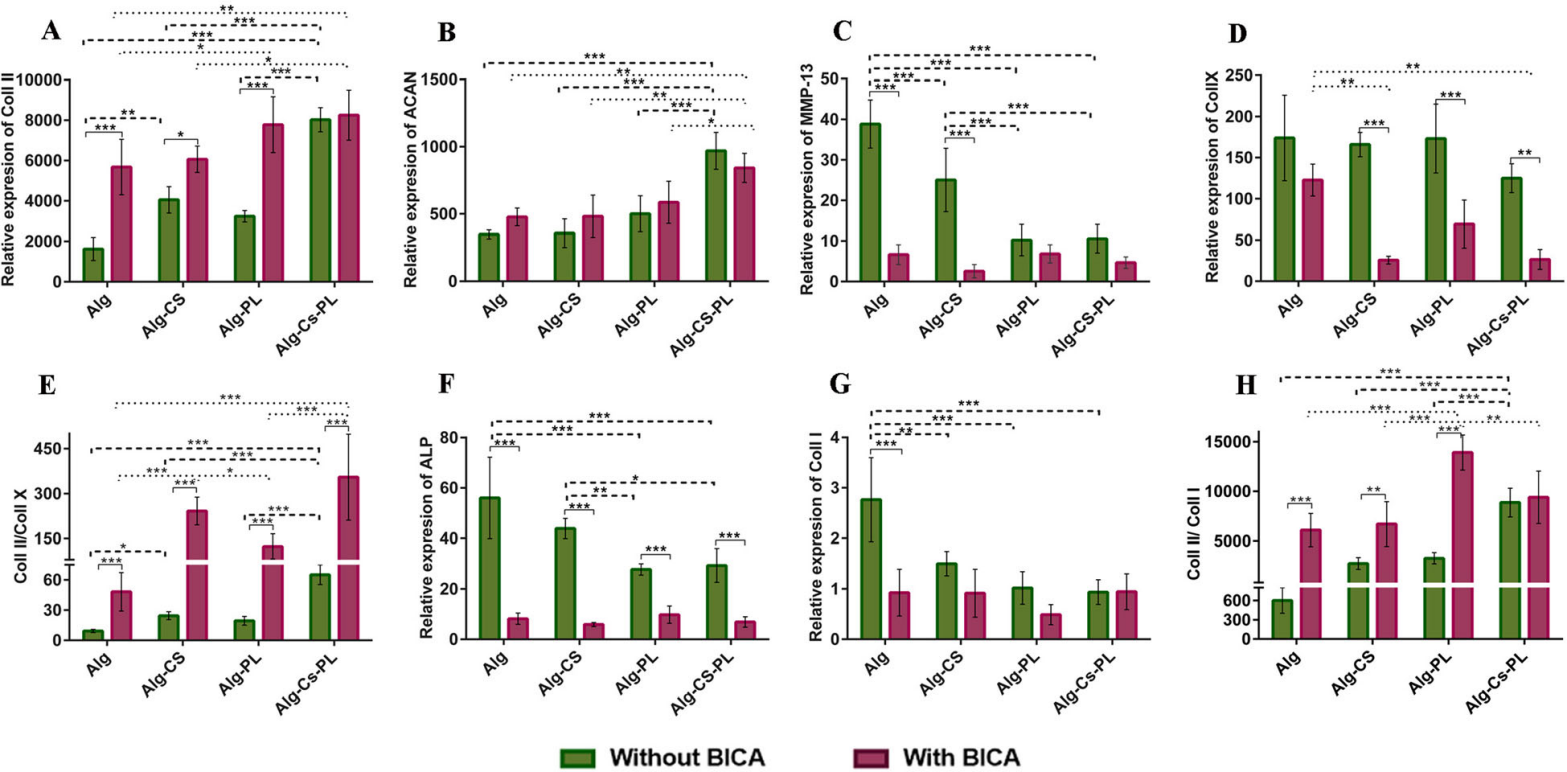
Chondrogenesis assessment in the presence or absence of 39 μM BICA. qRT-PCR was performed to investigate the expression of **a** Coll II, **b** ACAN, **c** MMP-13, **d** Coll X, **e** ratio between Coll II and Coll X, **f** ALP, **g** Coll I, and **h** ratio between Coll II and Coll I in four different hydrogels at day 28. Data are presented as means ± SD (*n* = 3) (*p < 0.05, **p < 0.01, ***p < 0.001)

In Fig. 7a, the expression of Coll II in Alg-CS-PL was significantly greater than all the other groups, when no BICA was present. Interestingly, in groups containing PL and BICA, Coll II expression was significantly higher compared to Alg- and Alg-CS groups. In all BICA-containing groups, except Alg-CS-PL, the expression of Coll II is significantly greater than the comparable groups without BICA.

The expression of ACAN in Alg-CS-PL (with or without BICA) was significantly higher than all other groups. It is worth mentioning that no significant difference was observed between groups with BICA in comparison to without BICA (Fig. 7b).

In the absence of BICA, the expression of MMP-13 in Alg-CS-PL and Alg-PL was significantly lower than Alg and Alg-CS groups (*p* < 0.001). Moreover, the expression of MMP-13 in Alg was significantly greater than Alg-CS (*p* < 0.001). A significant difference was also observed between Alg and Alg-CS groups with BICA compared with the same groups without BICA (*p* < 0.001). There was no significant difference between BICA-containing groups. (Fig 7c).

Coll X expression was similar in all groups without BICA. While Alg with BICA had similar expression of Coll X compared with BICA free samples, the addition of BICA to Alg-CS and Alg-CS-PL significantly reduced Coll X expression. BICA containing hydrogels demonstrated a clear inhibition of Coll X (Fig. 7d). When comparing Coll II/Coll X ratio, in order to assess stability of chondrogenic differentiation, in non-BICA groups, Alg-CS-PL had an increased ratio compared with other groups. Alg-CS also demonstrated a higher Coll II/Coll X ratio compared to Alg hydrogels. In BICA-containing hydrogels, the ratio of Coll II/Coll X in Alg-CS and Alg-CS-PL were significantly greater compared with the two other hydrogels. There was no significant difference between Alg-CS and Alg-CS-PL hydrogels. In addition, all hydrogels containing BICA showed a significant increase in Coll II/Coll X ratio compared to the same hydrogels without the small molecule (Fig. 7e).

The expression of ALP in Alg-PL and Alg-CS-PL, without BICA, was significantly lower than the other groups. No significant difference was observed among BICA-containing groups, although they showed significantly lower ALP expression compared to the groups without BICA (Fig. 7f).

The expression of Coll I in the Alg hydrogel without BICA was significantly higher than all the other groups. No significant difference was observed between the groups containing BICA (Fig. 7g). It was found that Coll II/ Coll I ratio in Alg-CS-PL hydrogel was significantly higher compared with other non-BICA hydrogels. In BICA-loaded groups, Alg-PL hydrogels had a greater Coll II/ Coll I ratio compared with other groups. There was also a significant difference in all BICA loaded gels except Alg-CS-PL when compared with the same hydrogel without BICA (Fig. 7h).

#### Histochemical assay and histological staining

**Fig. 8:**
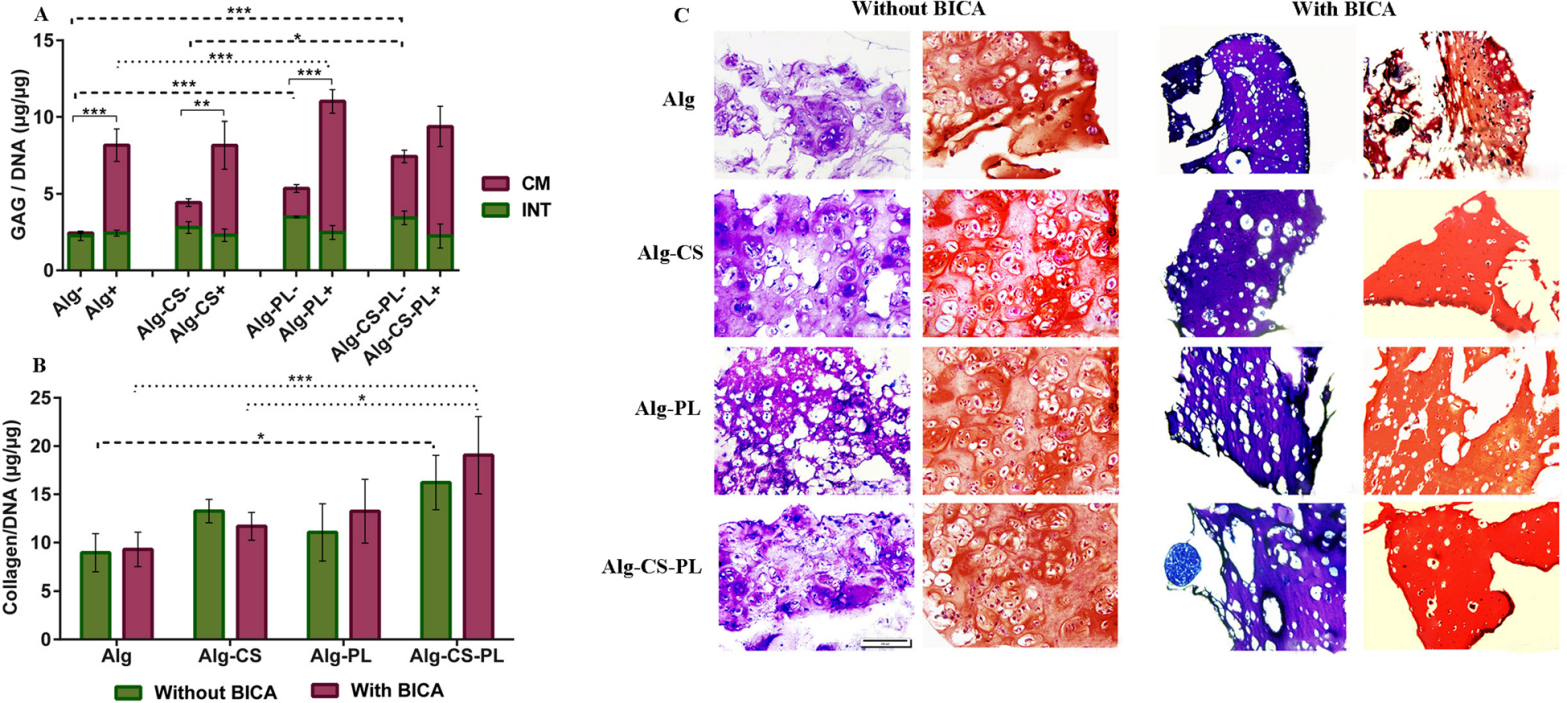
Biochemical and histological assessment of chondrogenic differentiation in the presence or absence of 39 μM BICA at day 28. **a** GAG produced by hBM-MSC-loaded hydrogels within conditioned media (CM) or within hydrogels (INT). **b** Total collagen content of hBM-MSC-loaded hydrogels. **c** Toluidine blue and Safranin-O & fast green staining. Data are presented as means ± SD (*n* = 3) (*p < 0.05, **p < 0.01, ***p < 0.001)

DMMB results revealed that GAG was retained within the hydrogel matrix but most GAG was released into the culture media. When BICA was absent, the GAG content for Alg-CS-PL was significantly greater than Alg-CS and Alg. The Total GAG content for BICA-containing groups was significantly higher than groups without BICA(*p* ≤ 0.01), except for the Alg-CS-PL group. When BICA was present, a significant difference was observed between Alg and Alg-PL (Fig. 8a) (*p* ≤ 0.05).

Although no significant differences were observed between BICA-containing and BICA-lacking groups, when BICA was present, the total collagen content in Alg-CS-PL hydrogel was significantly greater in comparison with Alg and Alg-CS (*p* ≤ 0.05). Alg-CS-PL without BICA also showed increased collagen production in comparison with Alg (*p* ≤ 0.05) (Fig. 8b).

Toluidine Blue and Safranin-O staining also demonstrated glycosaminoglycan deposition within the extracellular matrix by hBM-MSCs. In all conditions, there were a production of an abundant extra cellular matrix (ECM). (Fig. 8c).

### Mechanical loading

**Fig. 9:**
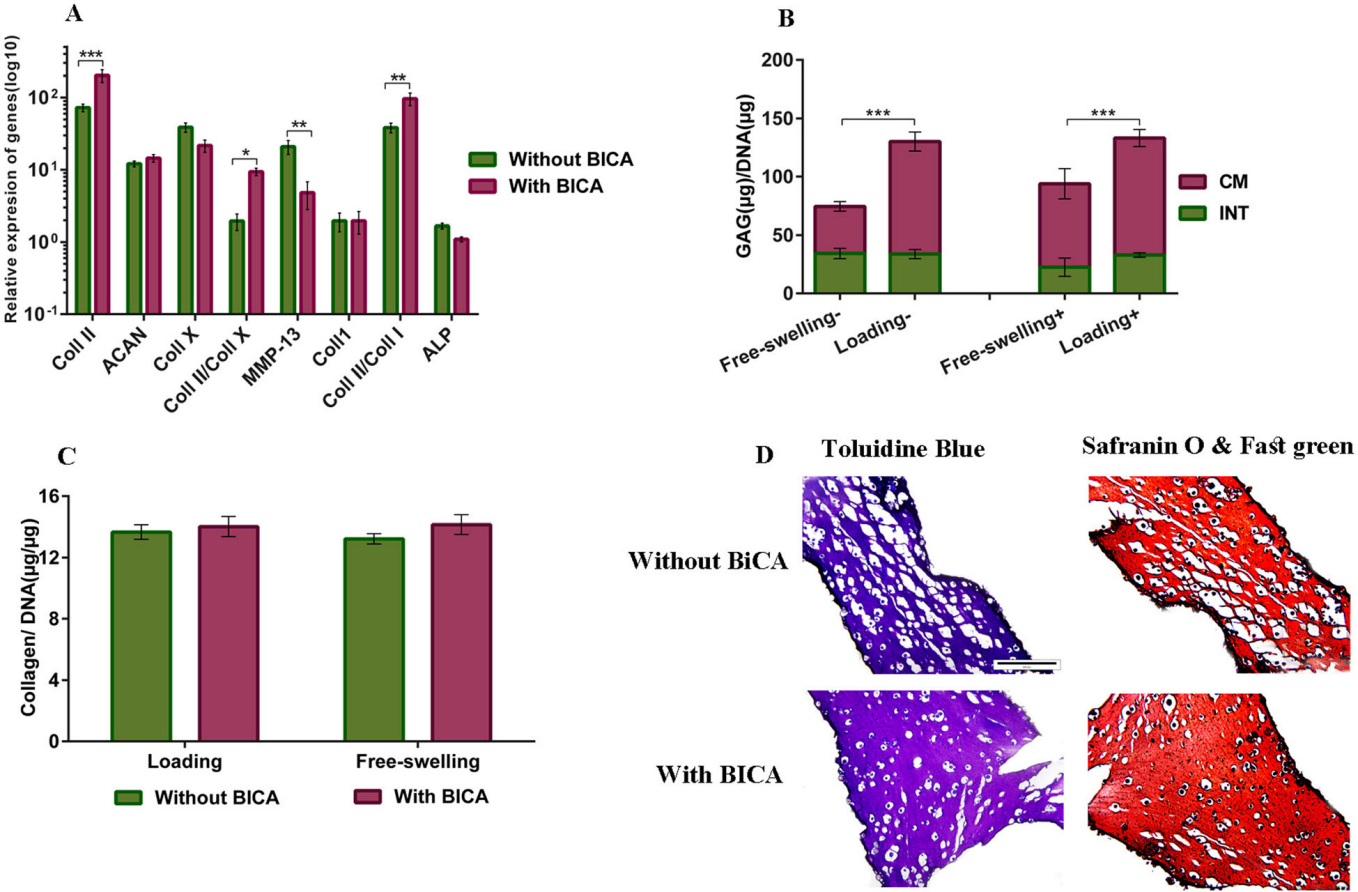
The effect of mechanical loading on chondrogenesis of hBM-MSCs in Alg-CS-PL hydrogels in the absence or presence of 39 μM BICA at day 28. **a** qRT-PCR of loaded samples normalized to unloaded samples. **b** GAG deposition of loaded and unloaded samples (−) without and (+) with BICA. **c** Collagen content of loaded and unloaded samples. **d** Toluidine blue and Safranin-O and fast green staining of loaded samples with and without BICA. Data are presented as means ± SD (*n* = 2) (*p < 0.05, **p < 0.01, ***p < 0.001)

The hydrogels containing the combination of Alg, CS, and PL (with or without BICA) were selected as the most appropriate hydrogel for investigation of mechanical loading, due to their better performance at the mRNA level, biochemical, and histological analysis. The effect of mechanical loading on the hydrogels is presented in Fig. 8. After mechanical loading, the expression of Coll II was significantly higher in the BICA-containing hydrogel (*p* ≤ 0.001). Furthermore, expression of the hypertrophy marker MMP-13 (*p* ≤ 0.01) was significantly lower in BICA-containing groups than in groups without BICA. The presence of BICA did not affect the expression of Coll I, ALP, and ACAN under mechanical loading. The ratio of Coll II/Coll X (*p* ≤ 0.05) and Coll II/Coll I (*p* ≤ 0.01) in BICA-loaded was significantly greater than non-BICA-loaded hydrogels. (Fig. 9a). Mechanical loading induced GAG production with or without BICA (Fig. 9b). Collagen secretion was not significantly affected, neither by the presence of BICA nor by mechanical loading. (Fig. 9c). Toluidine blue and Safranin-O staining showed that hBM-MSCs produced GAG with or without BICA. The images showed more coherent tissue in the presence of the BICA (Fig. 9d).

## Discussion

There are several key requirements regarding the properties of the ideal scaffold for tissue engineering: it should be biocompatible, non-cytotoxic, allow diffusion of nutrients, mechanically stable, readily available, and adaptable to be implanted in lesions of different sizes [35].

Alginate is extensively used in vitro, due to simple gelation mediated by Ca^2+^ ions [64, 65], and has been used for delivery of hMSCs in different tissue engineering applications such as cartilage [28, 66, 67]. However, Alg lacks a cell binding domain [68], and hMSCs are anchorage-dependent cells; their viability decreases if they cannot bind to matrix. Furthermore, Alg provides only a mechanical cue, with no biological cues such as growth factors or bioactive molecules; additionally, Alg is not a natural component of cartilage matrix.

Therefore, in the current study, PL, a potential autologous cocktail of proteins, growth factors, and anti-inflammatory cytokines, and CS, a main cartilage extracellular matrix component, were added to the hydrogel in order to develop a more favorable MSC delivery system for cartilage regeneration.

Our results showed that the Young’s modulus of Alg hydrogel increased by adding CS at a defined concentration (1% w/v). However, at higher concentration of CS, a decreased Young’s modulus was observed. Huang et al. indicated the incorporation of CS significantly increased the storage modulus of the Alg-foams [33].

The rheological measurements in accordance with the mechanical bulk testing showed that Alg hydrogel with 1% of CS had an increased viscoelastic property (G′ and G″) in comparison with Alg. CS molecules have a reinforcing effect, hypothetically attributed to an increase in polymer network density and water retention ability, without disruption of the Alg network. However, by increasing further the amount of CS within Alg hydrogel, a reduction of G′ and G″ is observed, likely related to a disruption of the ionic Alg crosslink network.

Combining CS with Alg increases the negative charge within the hydrogel network and influences the system arrangement by spatial occupation [69]. This potentially causes an increased space between polymer chains and decreases the mechanical properties of the hydrogels. Stuart and Panitch showed that CS incorporation to a collagen hydrogel decreased the viscoelastic properties of the collagen hydrogel due to changes in void space [70]. Furthermore, it was shown that the introduction of a negatively charged polysaccharide such as GAG into collagen hydrogels has different outcomes depending on the molecular weight, ratio of GAG to collagen gel, and ionic strength [71, 72].

We also added PL into the Alg hydrogel to improve the biological properties for cartilage tissue engineering. There have been several studies reporting PL to be a positive stimulus for repairing/regenerating different tissues, such as bone [73], skin [74], tendon [75], and cartilage [53]. However, two main problems remain: high degradability rate and low mechanical properties. Combination of PL in a hydrogel can protect growth factors and biomolecules from degradation and allow their continuous release [47]. Babo et al. incorporated PL into HA hydrogels which resulted in sustained release of growth factors and enhanced the viability of cells either encapsulated in or seeded within the hydrogels [76, 77]. Our results revealed that addition of PL increased the Young’s modulus as well as the viscoelastic modulus (G′, G″) of alginate hydrogel. It is possible that the clotting proteins of PL and the positive charges could enhance the alginate hydrogel crosslinking, resulting in a stronger network. In addition, the viscoelastic properties of the alginate gel with PL was higher than that of CS incorporated alginate gel.

It is important to consider that the cellular behavior is dependent on the hydrogel mechanical properties. Cells can reorganize and re-engineer their environments through mechanical and biochemical interaction with the substrate. Therefore, cells can strongly influence the hydrogel matrices in which they are embedded, in the short term through interaction with the crosslinking mechanism and long-term through remodeling [78–80].

There are few systematic studies that reported regarding changes in hydrogel mechanical properties upon addition of cells. For example, Buckley et al. showed increasing the cell seeding density effectively lowers the initial mechanical properties of a cell-seeded agarose hydrogel [81]. Our results showed embedding cells in Alg-based hydrogels does not change the Young’s modulus of the hydrogels.

Hu et al. demonstrated that Bortezomib, an FDA-approved small molecule for the treatment of myeloma, inhibits the expression of MMP-13, leading to a reduction of collagen type 2 degradation [82]. We based our initial screening range around a previous study that proposed 31 μM and 39 μm as suitable concentrations based on the structure of BICA and the Fragment-Based Screening study of Fragment-Based Discovery of Indole Inhibitors of Matrix Metalloproteinase-13 [83]. We then selected 39 μM based on our pellet-based screen. Interestingly, no significant difference was observed between groups in terms of cell viability.

Chondrogenic differentiation was evaluated for hBM-MSC pellets in the presence of BICA at different concentrations. Our data demonstrated a dose-dependent effect of BICA on hypertrophy and chondrogenesis of hBMCs. Hereafter BICA at a concentration of 39 μM was used for further studies.

Using a platform to deliver a drug directly to a lesion site may decrease the off-target effects associated with systemic drug injection by providing a prolonged but localized drug exposure [26, 27, 84]. An alginate-based hydrogel was used in this study to investigate BICA delivery during MSC chondrogenesis. In vitro release of BICA over nine consecutive days showed a similar trend for BICA release from the four hydrogels tested, with Alg-CS-PL having the lowest cumulative release compared to Alg-CS and Alg-PL.

The viability of hBM-MSCs encapsulated in Alg-based hydrogels was assessed. Previous studies have demonstrated the positive effect of PL upon the viability and proliferation of hBM-MSCs in combination with other hydrogels such as hyaluronic acid [47] and gellan gum gels [85], coating the scaffolds [86] and even used alone as a hydrogel [50]. In addition, it has been shown that incorporation of CS into the network supports the viability and proliferation of chondrocytes for at least 6 weeks [87]. Our results showed incorporation of PL and CS supported the viability of hBM-MSCs at day3, and 7. PL rescues the viability of hBM-MSCs by introduction of growth factors and CS as an enhancer of cell adhesion can protect cell proliferation and survival [88].

Chondrogenic differentiation of hBM-MSCs in the four different hydrogels was also studied. In the absence of BICA, expression levels of chondrogenic genes were significantly increased in Alg-CS-PL in comparison with other groups. Moreover, osteogenic genes such as ALP and Coll I and MMP-13 as a hypertrophy gene were also significantly decreased. This might be due to the synergistic effect of PL and CS on chondrogenic differentiation. As observed in previous studies, PL induces chondrogenic differentiation of adipose-derived [89] and umbilical cord-derived [52] MSCs. It has been shown that PL-loaded hyaluronic acid stimulates chondrogenesis of hMSCs [90]. Moreover, it was demonstrated chondroitin sulfate, an important molecule in cartilage extracellular matrix, improved chondrogenesis of MSCs [43]. Besides, Nguyen et al. demonstrated that PL induces reprograming of quiescent cartilage cells resulted in new cartilage formation [91].

The expression of Coll X along with MMP-13 is typically expressed in the hypertrophy pathway. Interestingly, unlike MMP-13, the results showed no effect the different hydrogels on Coll X expression. It is well-known that the expression of MMP-13 is controlled by different signaling pathways [92, 93]. One of the important mechanisms is the RUNX2 pathway that regulates both early (Coll X) and late (MMP-13) hypertrophic markers [94]. On the other hand, it has been demonstrated increased RUNX2 expression in articular chondrocytes may not be enough to increase MMP-13 expression [94]. In this study, the changes in MMP-13 and Coll X expression suggests PL regulates MMP-13 expression in a different way compared with Coll X, potentially in a Runx2-independent manner. However, this would require a more detailed analysis. Due to the transient and fluctuating nature of gene expression changes during differentiation, additional time points may be required to fully assess the gene expression changes.

Cell-hydrogel constructs were also used to study the effect of BICA on the inhibition of hBM-MSC hypertrophy.

When no BICA was present, GAG secretion in Alg-CS-PL showed the highest value which confirmed the mRNA results. Again, this suggests that PL and CS promoted chondrogenesis of hBM-MSCs. Previous studies have also indicated that CS can improve chondrogenic properties of hydrogels [43, 44]. In addition, Hildner at al. demonstrated GAG secretion of adipose-derived MSCs was induced in PL supplemented medium [89]. In BICA-loaded hydrogels, hypertrophic and osteogenic genes were downregulated, and at the same time, Coll II was upregulated. This suggests that BICA might be an appropriate inhibitory agent for hypertrophy. In the presence of BICA, the groups containing PL also showed the highest GAG secretion.

Histological staining was performed to detect the extracellular matrix deposition in the cell-laden hydrogels. The chondrogenic differentiation of hBM-MSCs was confirmed in both conditions, with and without BICA, by deposition of cartilage ECM and a spherical cellular phenotype.

Among the hydrogels with different combinations of Alg, CS, and PL, the hydrogel containing all three was selected as the most appropriate hydrogel due to its better performance, as assessed by mRNA, biochemical, and histological analysis. It has been shown that mechanical loading applied to MSCs induces chondrogenesis [95, 96]. It has also been demonstrated that shear and compressive load applied to MSCs embedded in fibrin-polyurethane scaffolds induced chondrogenesis [55–57]. Accordingly, complex load applied to hBM-MSCs in Alg-CS-PL was studied in the presence or absence of BICA. The expression level of MMP-13 confirmed the data previously obtained without the shear loading and indicated the effectiveness of BICA in inhibiting hypertrophy genes. Mechanical loading induced GAG production regardless of the presence of BICA, which confirmed the effect of mechanical loading on enhanced matrix synthesis. Total collagen secretion was not significantly affected by the mechanical loading, which suggests that the increase in collagen type 2 secretion opposed the decrease of collagen types 1 and 10. However, collagen types 1, 2, and 10 should be measured separately at post-translational level in order to confirm this.

## Conclusion

In conclusion, PL and CS enhance the mechanical properties and support cell viability in combination with Alg hydrogel. The results presented in this study showed that the tri-part hydrogel composed of Alg, CS, and PL can induce chondrogenic differentiation of hBM-MSCs by the upregulation of collagen type 2 and aggrecan. Also, it was shown that addition of BICA inhibited hypertrophy during chondrogenic differentiation of hBM-MSCs. Moreover, our results indicate that BICA improves hBM-MSCs differentiation into chondrocytes as it increases the expression level of chondrogenic genes as well as GAG and collagen deposition. In order to mimic the physiological environment of chondrocytes, a mechanical shear loading simulation was also provided. It is demonstrated that the applied loading regimen induces chondrogenic differentiation in case of GAG secretion.

## Supplementary information

**Additional file 1: Supplemental Table S1.** List of primers by using SyberGreen for qRT- PCR. **Supplemental Table S2**. Primer and probe sequences for qRT- PCR. **Supplemental Table S3**. List of assays on demand used for qRT-PCR.

**Additional file 2: S-Fig. 1**- Immunohistochemical staining against to Coll II and Coll X. **S-Fig. 2.** Flow cytometric analysis of CD markers in human BM-MSCs at passage 3. The majority of the cells expressed the typical CD markers related to MSC (CD 44 and CD 90, CD73, CD105). Other antigens were also expressed in a minority of the cells. **S-Fig. 3.** Differentiation potential of the isolated cells from human Bone Marrow. A) Sections prepared from micromass culture for chondrogenesis stained purple following toluidine blue staining, B) Osteogenic culture stained red following alizarin red staining. C) Adipogenic culture stained red following oil red staining. Culture conditions and staining methods were as previously described (Meury at al., Cell Biochem. 2006 Jul 1;98(4):992–1006).

## Data Availability

All data are included in the text and supplementary information.

## Abbreviations

BICA: 5-Bromoindole-2-carboxylic acid
CS: Chondroitin sulfate
PL: Platelet lysate
MMP-13: Matrix metalloproteinase-13
ECM: Extra cellular matrix
OA: Osteoarthritis
BM-MSCs: Bone marrow-derived mesenchymal stem cells
miRNAs: MicroRNAs
Alg: Alginate
FBS: Fetal bovine serum
GAG: Glycosaminoglycan
PCR: Polymerase chain reactions
INT: Intact
CM: Conditioned media
DMMB: 1,9-Dimethyl-methylene blue
qPCR: Quantitative polymerase chain reaction
