# Supplementary material for: Inhibition of hypertrophy and improving chondrocyte differentiation by MMP-13 inhibitor small molecule encapsulated in alginate-chondroitin sulfate-platelet lysate hydrogel
Source: Stem Cell Res Ther. 2020 Oct 9;11:436. doi: 10.1186/s13287-020-01930-1 (PMC7545577; doi:10.1186/s13287-020-01930-1)
Supplement: Supplementary file 1 — Additional file 1: Supplemental Table S1. List of primers by using SyberGreen for qRT- PCR. Supplemental Table S2. Primer and probe sequences for qRT- PCR. Supplemental Table S3. List of assays on demand used for qRT-PCR. [file 13287_2020_1930_MOESM1_ESM.docx]

**Inhibition of Hypertrophy and Improving Chondrocyte Differentiation by MMP-13 Inhibitor Small Molecule-Encapsulated in Alginate-Chondroitin Sulfate-Platelet Lysate Hydrogel**

Shahrbanoo Jahangir^1^, David Eglin^2^, Naomi Pötter^3,2^, Mojtaba Khozaei Ravari^4^, Martin Stoddart^2^, Ali Samadikuchaksaraei^5,6,1^, Mauro Alini^2, *^, Mohammadreza Baghaban Eslaminejad^4, *^, Majid Safa^5,^ ^7,1, *^

Supplemental Table S1: list of primers by using SyberGreen for qRT- PCR.

| **β-actin** | F: 5' CTC CTC TCA AAC CCA AAC TC 3' |
| --- | --- |
|  | R: 5' GTC CTC AAC AAA CAC CAT CAC 3' |
| **MMP-13** | F: 5'TCCTGATGTGGGTGAATACAATG3' |
|  | R: 5'GCCATCGTGAAGTCTGGTAAAAT3' |
| **Coll X** | F:5' GAA CGA TAC CAA ATG CCC AC 3' |
|  | R:5' GAC GAC CAG GAG CAC CAT AT 3' |

Supplemental Table S2. Primer and probe sequences for qRT- PCR.

| **Gene** | **Forward primer** | **Reverse primer** | **Probe** |
| --- | --- | --- | --- |
| *Coll II* | 5' – GGC AAT AGC AGG TTC ACG TAC A -3' | 5'- GAT AAC AGT CTT GCC CCA CTT ACC -3' | 5'- CCT GAA GGA TGG CTG CAC GAA ACA TAC -3' |
| *Coll X* | 5'- ACG CTG AAC GAT ACC AAA TG -3' | 5'- TGC TAT ACC TTT ACT CTT TAT GGT GTA -3' | 5'- ACT ACC CAA CAC CAA GAC ACA GTT CTT CAT TCC -3' |
| *MMP13* | 5'- CGG CCA CTC CTT AGG TCT TG -3' | 5'- TTT TGC CGG TGT AGG TGT AGA TAG -3' | 5'- CTC CAA GGA CCC TGG AGC ACT CAT GT -3' |
| *Coll I* | 5'- CCC TGG AAA GAA TGG AGA TGA T -3' | 5'- ACT GAA ACC TCT GTG TCC CTT CA -3' | 5'- CGG GCA ATC CTC GAG CAC CCT -3' |
| *ACAN* | 5'- AGT CCT CAA GCC TCC TGT ACT CA -3' | 5'- CGG GAA GTG GCG GTA ACA -3' | 5'- CCG GAA TGG AAA CGT GAA TCA GAA TCA ACT -3' |

Supplemental Table S3. List of assays on demand used for qRT-PCR.

| Gene | Assay ID |
| --- | --- |
| Human 18S | Hs99999901_s1 |
| Human ALPL | Hs01029144_m1 |
