# Supplementary material for: Inhibition of hypertrophy and improving chondrocyte differentiation by MMP-13 inhibitor small molecule encapsulated in alginate-chondroitin sulfate-platelet lysate hydrogel
Source: Stem Cell Res Ther. 2020 Oct 9;11:436. doi: 10.1186/s13287-020-01930-1 (PMC7545577; doi:10.1186/s13287-020-01930-1)
Supplement: Supplementary file 2 — Additional file 2: S-Fig. 1- Immunohistochemical staining against to Coll II and Coll X. S-Fig. 2. Flow cytometric analysis of CD markers in human BM-MSCs at passage 3. The majority of the cells expressed the typical CD markers related to MSC (CD 44 and CD 90, CD73, CD105). Other antigens were also expressed in a minority of the cells. S-Fig. 3. Differentiation potential of the isolated cells from human Bone Marrow. A) Sections prepared from micromass culture for chondrogenesis stained purple following toluidine blue staining, B) Osteogenic culture stained red following alizarin red staining. C) Adipogenic culture stained red following oil red staining. Culture conditions and staining methods were as previously described (Meury at al., Cell Biochem. 2006 Jul 1;98(4):992–1006). [file 13287_2020_1930_MOESM2_ESM.docx]

**Inhibition of Hypertrophy and Improving Chondrocyte Differentiation by MMP-13 Inhibitor Small Molecule-Encapsulated in Alginate-Chondroitin Sulfate-Platelet Lysate Hydrogel**

Shahrbanoo Jahangir^1^, David Eglin^2^, Naomi Pötter^3,2^, Mojtaba Khozaei Ravari^4^, Martin Stoddart^2^, Ali Samadikuchaksaraei^5,6,1^, Mauro Alini^2, *^, Mohammadreza Baghaban Eslaminejad^4, *^, Majid Safa^5,^ ^7,1, *^


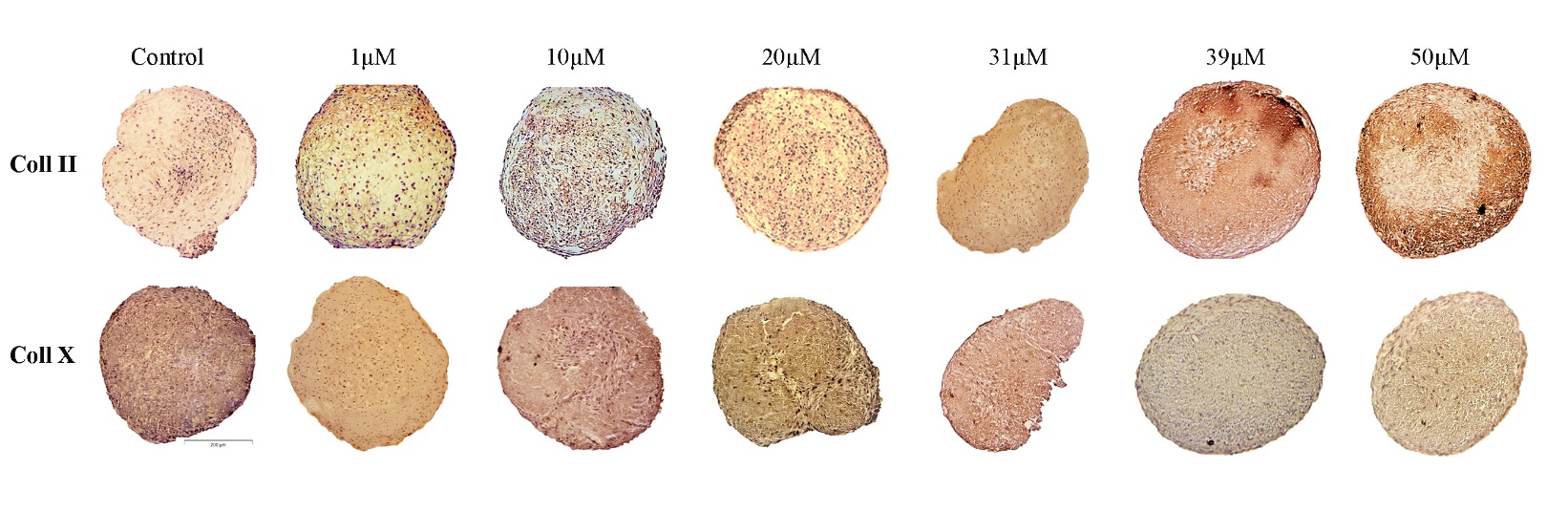
S-fig1- Immunohistochemical staining against to Coll II and Coll X.


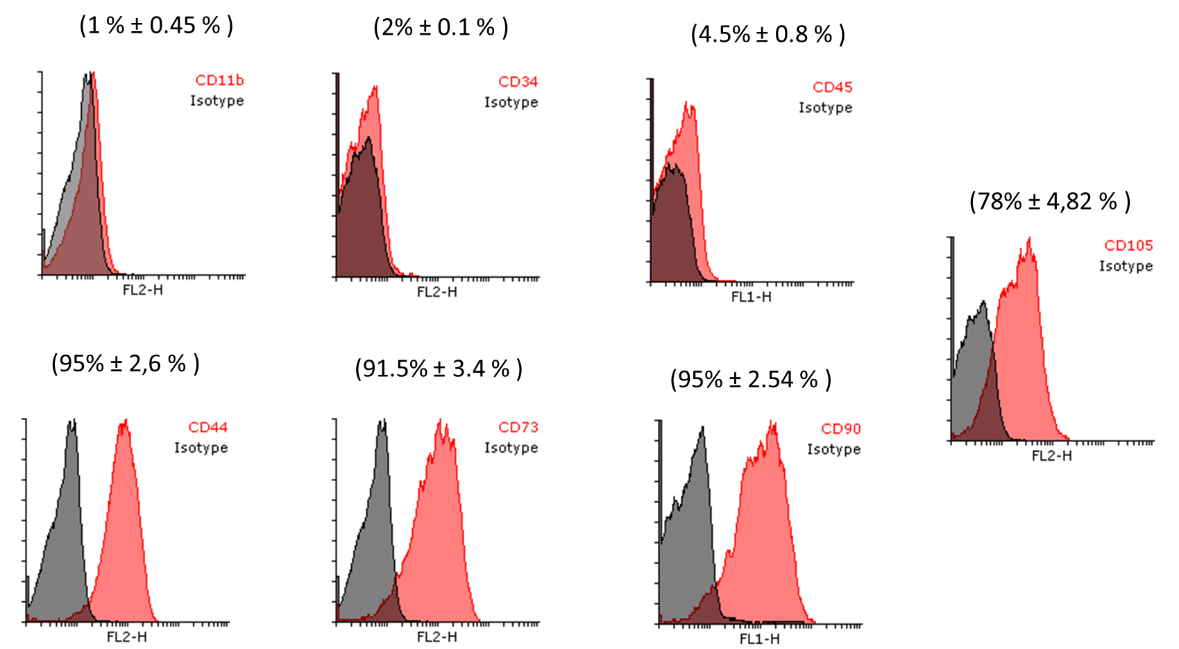


S-fig 2: Flow cytometric analysis of CD markers in human BM-MSCs at passage 3. The majority of the cells expressed the typical CD markers related to MSC (CD 44 and CD 90, CD73, CD105). Other antigens were also expressed in a minority of the cells.


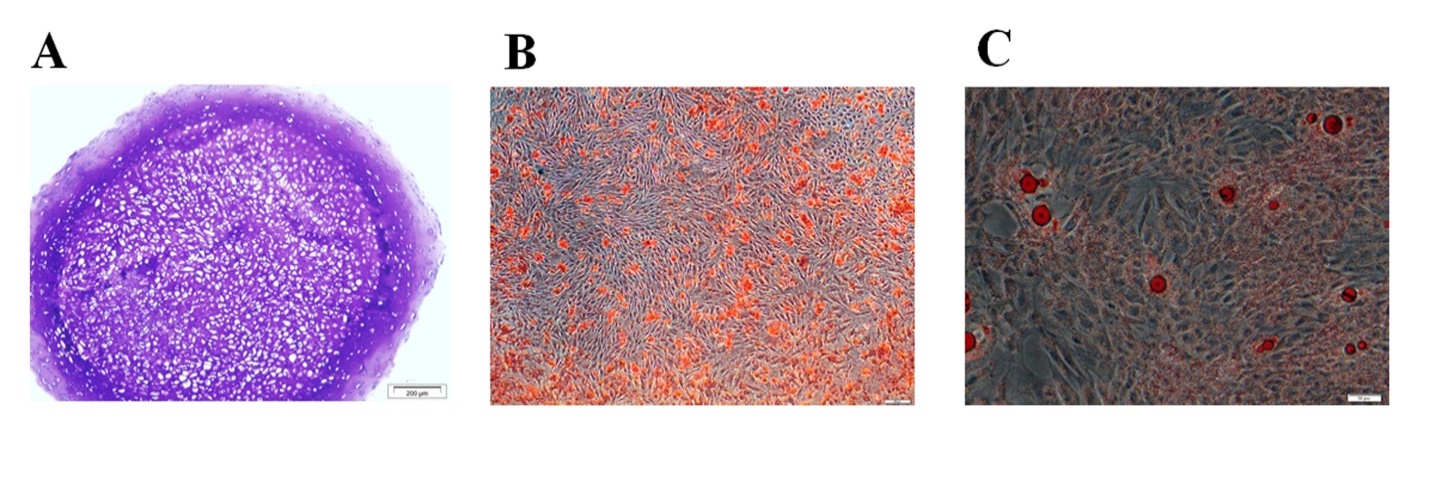
S-fig3: Differentiation potential of the isolated cells from human Bone Marrow. A) Sections prepared from micromass culture for chondrogenesis stained purple following toluidine blue staining, B) Osteogenic culture stained red following alizarin red staining. C) Adipogenic culture stained red following oil red staining. Culture conditions and staining methods were as previously described (Meury at al., [Cell Biochem.](https://www.ncbi.nlm.nih.gov/pubmed/16479590) 2006 Jul 1;98(4):992-1006)
